# Aberrant cortico-striato-limbic functional connectivity to alcohol cues in co-occurring bipolar disorder and alcohol use disorder

**DOI:** 10.1038/s41386-026-02418-x

**Published:** 2026-05-02

**Authors:** William H. Mellick, Bryan K. Tolliver, Raymond F. Anton, James J. Prisciandaro

**Affiliations:** Addiction Sciences Division, Department of Psychiatry and Behavioral Sciences, Medical University of South Carolina, Charleston, SC USA

**Keywords:** Reward, Human behaviour, Diagnostic markers, Motivation, Classical conditioning

## Abstract

Reward brain circuitry dysfunction is a hypothesized mechanism of bipolar disorder and alcohol use disorder co-occurrence (BD + AUD) that remains largely untested. This neuroimaging study represents the first investigation of functional connectivity in BD + AUD. Following a two-by-two factorial design (*N* = 90), individuals with BD + AUD (*n* = 22), AUD alone (*n* = 20), BD alone (*n* = 23), and healthy control participants (*n* = 25) were administered a fMRI alcohol-cue reactivity paradigm. Generalized psychophysiological interaction (PPI) modeling (*p* < 0.001; *p*-FDR < 0.05) was performed for regions of interest, including the right dorsal anterior insula, inferior frontal gyrus, and bilateral amygdala and dorsal striatum (i.e., caudate body). Extracted beta weights were explored for bivariate associations with key behavioral correlates (AUD age of onset, alcohol craving and dependence severity, abstinence duration, and impulsivity) (*p* < 0.05). BD + AUD individuals exhibited cue-modulated hyperconnectivity between the left dorsal striatum and right posterior cingulate cortex (*p*-FDR = 0.045) versus the AUD and BD groups, who both exhibited hypoconnectivity between these regions versus healthy participants. Additionally, there were main effects of AUD and BD (*p*-FDR ≤ 0.040) on cue-modulated functional connectivity of the right dAI (↓ middle frontal gyrus [MFG]) and left amygdala (↑ right superior temporal gyrus, anterior cingulate cortex, and MFG), respectively. Select functional connectivity data were associated with trait characteristics of AUD in BD + AUD (*r* ≥±0.50, *p* ≤ 0.026) but not AUD. A distinct pattern of cortico-striato-limbic functional connectivity and brain-behavior relationships was found to characterize BD + AUD with implications for treatment development. Namely, leveraging neuromodulation techniques that can effectively normalize the identified circuitry disruptions could represent a novel path for treatment advances in BD + AUD.

## Introduction

Nearly half of individuals with bipolar disorder (BD) develop co-occurring alcohol use disorder (AUD), leading to more severe manic and depressive symptoms, more frequent mood episodes, treatment resistance, and increased suicide risk, disproportionately contributing to the $195 billion estimated total annual national economic burden associated with BD [1, 2]. The neurobiology underlying co-occurring BD and AUD (BD + AUD) remains poorly understood because, historically, AUD and BD have been studied independently. This approach has resulted in a significant knowledge gap that has hindered the identification of clearly effective treatments for patients with BD + AUD [3, 4]. Investigating shared neural mechanisms of BD and AUD, namely reward brain circuitry dysfunction, is a promising strategy for improved understanding and advanced treatment for BD + AUD [5–7]. Both disorders are thought to emerge from anomalous reward processing functional connectivity [8, 9], yet surprisingly few clinical neuroimaging studies to date have been conducted in either disorder. Towards this end, in the present study, we specifically sought to elucidate aberrant alcohol reward-modulated functional connectivity in BD + AUD towards the identification of novel network-level disruptions that can, in turn, guide treatment development efforts.

Reward processing recruits an interconnected, dopamine-rich brain network comprised of an expansive set of brain regions embedded along the meso-cortico-striato-limbic pathway stemming from the midbrain ventral tegmental area [10]. With well-defined functional roles, the rewarding and motivational properties of environmental stimuli are encoded by subcortical regions, including the striatum (reward detection [ventral]; motor initiation [dorsal]) and amygdala (affective/motivational salience attribution), that target cortical regions for the coordination and execution of higher-level processing including goal representation (e.g., ventromedial prefrontal cortex), action planning (e.g., posterior cingulate cortex [PCC]), conflict monitoring (e.g., anterior cingulate cortices [ACC]), and motivated decision-making (e.g., right inferior frontal gyrus [IFG]/anterior insula) [11–13]. Independently, BD and AUD have each been found to involve a dynamic dysregulation of the reward and cognitive control brain circuitry that mediates incentive salience and motivated behavior [14, 15].

AUD is commonly marked by compulsive drinking and alcohol-seeking behavior, a lack of control over drinking, and distressing negative emotional withdrawal states [16]. In the development and progression of AUD, chronic heavy drinking induces neuroadaptations of dopamine-modulating cortico-striatal glutamatergic pathways that consequently affect the functioning of reward and learning brain circuitry. Namely, preclinical/animal models and human functional neuroimaging research have shown there to be a cardinal shift in reward processing from the ventral to dorsal striatum, and a functional imbalance that emerges between prefrontal and insula circuits along with impaired recruitment of the IFG (i.e., the “brake” that suppresses maladaptive (stimulus-)responding [17]) in individuals with AUD—corresponding with habit formation, compulsivity, and the prioritization of behavioral repertoires that promote the pursuit of alcohol and alcohol-conditioned stimuli (cues) over alternative rewards (i.e., behavioral inflexibility) [18, 19]. Following suit, alcohol-cue processing has received considerable attention in the alcohol neuroimaging literature, although surprisingly few studies have examined cue-modulated (i.e., task-based) functional connectivity, outside of clinical trials where findings were obscured by treatment medications. Meta-analytic findings of reward network connectivity across substance use disorders (SUDs), however, suggest that aberrant cue processing in AUD involves a pattern of reduced functional connectivity (hypoconnectivity) between the ventral striatum and PCC, and between the dorsal striatum and principal nodes of the salience network (i.e., the insula and ACC) [20]. Consistently, relevant studies of individuals with AUD, specifically, have found reward-modulated fronto-striatal hypoconnectivity to be distinguishing and associated with trait characteristics (e.g., obsessive craving, choice impulsivity, and alcohol use severity) [20, 21].

Core features of BD, including reward hypersensitivity, emotional lability, and emotion dysregulation, have been found by functional neuroimaging studies to reflect abnormalities in fronto-cortico-striatal (reward) and fronto-cortico-amygdala (emotion regulation) brain circuity irrespective of mood state [22]. In response to a range of appetitive/rewarding stimuli across environmental contexts, individuals with BD have exhibited functional hyperactivation of the amygdala, striatum, and medial prefrontal brain regions, suggesting a generally heightened perception of incentive and emotional salience in BD [7, 23, 24]. Limited research has investigated the underlying functional connectivity of this neurocircuitry. Extant findings, focused on delayed reward decision-making (i.e., delay discounting) and reward feedback processes, have characterized BD by ventral striatal hypoconnectivity with the anterior prefrontal cortex (i.e., middle frontal gyrus) in both instances and cortico-striato-amygdala hyperconnectivity (e.g., striatum ↔ amygdala, anterior insula) during reward feedback, specifically. These circuit disruptions, in turn, were associated with (hypo)manic symptoms and impulsivity measures across studies [24, 25]. Taken together, hyper-engagement (i.e., ↑ functional coupling) of the amygdala in response to rewarding stimuli is seemingly specific to BD, whereas ineffective top-down modulation of mesolimbic reward dopamine signaling (e.g., via IFG hypo-engagement) and choice impulsivity mechanisms dually contribute to reward dysfunction in BD and AUD, respectively [25, 26].

Previously, our research group conducted the first functional neuroimaging reward processing study of BD + AUD [27]. Using a two-by-two factorial (BD × AUD) design with a well-established alcohol-cue reactivity paradigm [28], we found BD + AUD individuals, relative to participants with AUD or BD and healthy control participants, to be uniquely characterized by cue-elicited hypoactivations within the dorsal striatum (i.e., caudate body) and right dorsal anterior insula (dAI) and adjacent IFG, which, in turn, were associated with obsessive-compulsive craving and impulsivity-related features in BD + AUD. The current research sought to extend these initial findings, using the same data to examine whether the cue-modulated functional connectivity of our identified regions of interest (ROIs; the right dAI and IFG and bilateral caudate body), with the addition of the bilateral amygdala given its roles in associative learning, emotion dysregulation and relapse vulnerability [29–34], may also distinguish individuals with BD + AUD [34–37]. We broadly hypothesized that cue-modulated dorsal striatal hypoconnectivity with frontal and midline brain regions (e.g., insula, PCC), along with amygdala hyperconnectivity, would be found to characterize BD + AUD and that distinct brain-behavior relationships with our correlates of interest (characteristics of AUD [e.g., AUD age of onset, alcohol craving and dependence severity], impulsivity [including maximum number of drinks on a single occasion], and duration of abstinence) would be present in BD + AUD but not in other participant groups.

## Materials and methods

### Participants

One-hundred and twelve participants were enrolled into one of four groups based on whether they met Diagnostic and Statistical Manual of Mental Disorders, Fourth Edition, Text Revision (DSM-IV-TR; [38]) criteria for BD and/or alcohol dependence (approximating moderate-to-severe AUD in the DSM-5 [39], herein referred to as AUD): BD + AUD, AUD alone, BD alone, or healthy control (HC) participants free from psychiatric disorder. At minimum, participants were required to be between 18 and 65 years of age, capable of consenting and completing study procedures, and able to abstain from alcohol and drugs for ≥1 week (verified by serial biomarker testing) before MRI scanning to avoid potential confounding effects of intoxication or withdrawal [40]. General exclusion criteria included serious medical illness; clinically-significant head injury; past-month electroconvulsive therapy; psychotic disorder; recurrent major depressive disorder; past-month posttraumatic stress disorder, obsessive-compulsive disorder or eating disorder; current benzodiazepine or antidipsotropic use (e.g., naltrexone, acamprosate, disulfiram); history of delirium tremens, alcohol withdrawal seizures, or significant alcohol withdrawal (>7 on the Clinical Interview for Withdrawal Assessment for Alcohol—Revised; CIWA-Ar [41]) and/or medical detoxification within 2 weeks of MRI scanning; claustrophobia; or MRI incompatible surgical implants or (non)ferrous materials.

Group-specific inclusion/exclusion criteria required BD + AUD and AUD participants to meet DSM-IV-TR criteria for current (past 3-month) alcohol dependence (i.e., moderate-to-severe AUD per the DSM-5) and exceed NIAAA-defined heavy drinking levels (i.e., >14 [men] or >7 [women] alcohol drinks/week) [42] (on average) in the month before enrollment or, if abstinent at intake, the month preceding their last drink of alcohol (i.e., because inpatient referrals were coming from a controlled environment). BD + AUD and AUD participants with co-occurring (past 3-month) drug dependence (approximating moderate-to-severe SUD) were also included for feasibility and generalizability purposes. Psychotropic medications were permitted for BD + AUD and BD participants, but additions, discontinuations, or dose changes >20% made within 1 week prior to MRI scanning were exclusionary [43]. Finally, exceeding NIAAA-defined heavy drinking levels, reporting frequent (> weekly) past-month drug use, or meeting criteria for any non-tobacco-related SUD excluded potential BD and HC participants.

### Procedures

This study was conducted at the Medical University of South Carolina following institutional review board approval. All participants provided written informed consent. Licensed study clinicians primarily used the Structured Clinical Interview for DSM-IV Axis I Disorders (SCID-IV-TR; [44]), a 90-day alcohol/substance use timeline followback (TLFB; [45]), the CIWA-Ar [41], and biospecimen lab results to determine eligibility. Past-week mood symptoms were clinician-rated using the Montgomery-Asberg Depression Rating Scale (MADRS; [46]) and Young Mania Rating Scale (YMRS; [47]). Self-report questionnaires included the Alcohol Dependence Scale (ADS; [48]), Obsessive-Compulsive Drinking Scale (OCDS; [49]), and Barratt Impulsiveness Scale (BIS-11; [50]). MRI scanning was completed approximately 4 days following abstinence verification, after TLFB data were updated and the MADRS, YMRS, and OCDS were re-administered.

#### Abstinence verification

Required abstinence was monitored using a multimodal approach. Urine testing for ethyl glucuronide (EtG) [51], an ethanol metabolite detectable within 24–72 h of consumption (200 ng/mL threshold), and drug screening (UDS) corroborated self-report. Participants had two opportunities to demonstrate abstinence before exclusion. The long half-life of cannabis could create conflicting TFLB and cannabinoid-UDS data [52], which, under any circumstance, excluded BD and healthy control participants. For BD + AUD and AUD participants, specifically, a validated algorithm distinguished new cannabis use from residual cannabinoid excretion [53]. Five participants whose positive cannabinoid-urine drug screening was due to residual cannabinoid excretion were retained. Each and every participant included in the analyzable sample demonstrated ≥1 week of abstinence via serial self-report and drug and alcohol biomarker testing.

### MRI

#### Alcohol-cue reactivity paradigm

This well-validated fMRI paradigm, used by our team over the past 20 years [27, 54], reliably elicits robust reward neurocircuitry activation via presentation of pseudorandomly interspersed images of alcohol (ALC) and non-alcohol (BEV) beverages, blurry control images, and fixation (Fig. S1). Participants’ urge for alcohol was rated by handpad between blocks, providing a 6 s washout period for the hemodynamic response to return to baseline.

#### Data processing

MRI data were fully processed and analyzed using the CONN functional connectivity toolbox [55] in SPM, version 12 (UCL Queen Square Institute of Neurology). Functional and anatomical data preprocessing included realignment with susceptibility distortion correction using field maps, slice timing correction, outlier detection, direct segmentation, normalization into Montreal Neurological Institute space, and 8-mm smoothing. Potential outlier brain volumes were identified using ART [56] (framewise displacement ≥0.9 mm or global BOLD signal changes ≥5 standard deviations) [57]. For details on our denoising and quality control procedures, see Supplementary Materials.

### Data analytic strategy

#### First-level analysis

Generalized psychophysiological interaction (gPPI) modeling was performed to study functional connectivity changes across ALC and BEV conditions. Following our published cue reactivity findings in BD + AUD [24] and guiding literature, we selected four ROIs using the cross-validated, connectivity-based Brainnetome Atlas (http://atlas.brainnetome.org) for seed-based gPPI analyses [58]: the right dAI ([regions] 168 + 179), right IFG (pars opercularis; 38), the bilateral caudate body (227 and 228), and bilateral amygdala (211 and 212). Separately for each seed, a gPPI model [59, 60] was defined with seed BOLD signals as physiological factors, boxcar signals characterizing each task condition convolved with a canonical hemodynamic response function as psychological factors, and the product of the two as psychophysiological interaction terms. The multivariate regression coefficient of the psychophysiological interaction terms in each model designated task-modulated changes in functional connectivity.

#### Group-level analysis

For each individual voxel, a separate two-by-two general linear model (GLM) was estimated, with first-level connectivity measures at this voxel as dependent variables, and BD (0,1), AUD (0,1), and the BD × AUD interaction term as independent variables. gPPI results were thresholded using a standard combination of a cluster-forming *p* < 0.001 voxel-level threshold, and a familywise corrected *p*-FDR < 0.05 cluster-size threshold [61]. Cluster-level inferences were based on parametric statistics from Gaussian Random Field theory [55, 62]. Potential covariates, objectively identified a priori, were evaluated using an established model-building approach whereby primary analyses were performed with and without potential covariate adjustment, retaining only statistically significant covariates, to identify final parsimonious models [27, 40, 63].

SPSS, version 30 (IBM), was used to analyze sociodemographic and clinical variables pertaining to all subgroups using two-by-two GLMs when continuous (*F*) or GLMs with a logit link when dichotomous (*Wald*). Group-inclusive variables were analyzed among relevant subgroups using independent samples *t-*tests when continuous or chi-square (*χ* ^2^) tests of independence when dichotomous. Effect sizes were reported in *η*^2^ or Cohen’s *d*, respectively. Associations between gPPI data and behavioral correlates of interest were explored using Pearson bivariate correlations (*r*) (*p* < 0.05), knowing that we were statistically underpowered to evaluate moderation models [64].

## Results

### Sample characteristics

Of the overall sample (*N* = 112), 22 participants were removed from analyses following denoising and quality control procedures (see Supplementary Materials). Across the final sample (*N* = 90), participants self-identified as White (81.8%, *n* = 72) or Black/African American (14.8%, *n* = 13), with one participant each self-identifying as either Asian, Native American, or Pacific Islander, or Hispanic. The average age was 37.53 years (SD = 11.34), 50.0% (*n* = 45) were female, 27.3% (*n* = 24) were smokers (defined as ≥10 cigarettes/day; [65]), 70.2% (*n* = 59) reported drinking alcohol within the prior month, and 4.4% (*n* = 4) reported drug use within 2 weeks of MRI scanning. The BD + AUD and AUD groups were strictly matched in terms of self-reported AUD severity per the ADS (*p* = 0.357), as well as diagnostically in terms of average number of DSM-IV-TR criteria met (*p* = 0.176) and the assigned severity category (i.e., moderate versus severe; *p* = 0.168). Across AUD groups, only 4 participants met drug dependence criteria within 1 month of baseline (BD + AUD, cannabis [*n* = 2] or cannabis/stimulant [*n* = 1]; AUD, cocaine [*n* = 1]). Fourteen participants (BD + AUD, *n* = 5; BD, *n* = 1; AUD, *n* = 4; HC, *n* = 4) required an intermediate visit(s) for abstinence verification. For further sample characteristics, see Table 1 and Fig. S1.

**Table 1:** Sample characteristics and group comparison results.

	BD + AUD^1^ (*n* = 22)	AUD^2^ (*n* = 20)	BD^3^ (*n* = 23)	HC^4^ (*n* = 25)	*F*/Wald (*p*)		
					AUD	BD	BD × AUD
Age (years)^a^	36.65 (11.66)	42.45 (12.94)	34.30 (10.68)	37.28 (9.51)	2.48 (0.119)	3.37 (0.070)	0.35 (0.556)
Sex (%female [*n*])^a^	50.00 (10)	35.0 (7)	60.9 (14)	52.0 (13)	1.71 (0.191)	1.26 (0.261)	0.09 (0.768)
Smoking status (%yes [*n*])	40.00 (8)	25.0 (5)	17.4 (4)	28.0 (7)	1.02 (0.312)	0.01 (0.936)	1.75 (0.186)
YMRS^b^	2.10 (2.86)^4^	1.10 (1.41)	1.36 (1.94)	0.36 (0.81)^1^	3.38 (0.069)	6.23 (0.015)	0.00 (0.996)
MADRS^b^	10.50 (10.22)^2,4^	3.90 (4.10)^1^	7.41 (6.27)^4^	1.00 (1.94)^1,3^	4.99 (0.028)	23.54 (<0.001)	0.01 (0.943)
ADS^c^	19.00 (6.94)^3,4^	16.70 (8.58)^3,4^	2.09 (2.15)^1,2^	0.72 (1.34)^1,2^	201.39 (<0.001)	2.51 (0.117)	0.16 (0.689)
Drank ≤1 month (%yes [*n*])	83.30 (15)	63.2 (12)	54.5 (12)^d^	80.0 (20)	0.32 (0.573)	0.02 (0.897)	4.88 (0.027)
**Group-inclusive variables**						***χ*/*t*** **(*p*)**	
BD subtype (%Type-I [*n*])	70.00 (14)	-	56.50 (13)	-		0.83 (0.362)	
BD onset (years)	27.16 (8.93)	-	27.00 (9.52)	-		0.51 (0.612)	
AUD onset (years)	23.16 (8.93)	26.15 (6.00)	-	-		1.43 (0.162)	
AUD treatment (%yes [*n*])	45.50 (10)	65.00 (13)	-	-		0.92 (0.337)	
Drug Dependence (%yes [*n*])	20.00 (4)	25.00 (5)	-	-		0.14 (0.999)	
Anxiety Disorder (%yes [*n*])	50.50 (11)^2^	15.00 (3)	47.80 (11)^2^	-		7.69 (0.021)	
Medication (%yes [*n*])							
Lithium	15.00 (3)	-	21.70 (5)	-		0.32 (0.704)	
Antipsychotic	30.00 (6)^3^	-	60.90 (14)^1^	-		4.10 (0.043)	
Anticonvulsant	65.00 (13)	-	60.00 (12)	-		1.28 (0.258)	
Antidepressant	60.00 (12)	-	34.80 (8)	-		2.73 (0.098)	

Data are mean (S.D.) and *F* or Wald test statistic (*p* value), respectively.

“-” denotes variables that did not apply to one or more groups by design. Smoking status is defined as ≥10 cigarettes/day; Drug dependence and Anxiety disorder (%) refer to DSM-IV-TR diagnostic criteria being met within the past 3 months.

Superscripts^1-4^ assigned to descriptive statistics denote significant group differences (uncorrected *p* < 0.05).

*AUD* alcohol use disorder, *BD* bipolar disorder, *YMRS* Young Mania Rating Scale, *MADRS* Montgomery-Asberg Depression Rating Scale, *ADS* Alcohol Dependence Scale.

^a^Matching variable for all groups.

^b^Matching variable for BD + AUD and BD alone groups.

^c^Matching variable for BD + AUD and AUD alone groups.

^d^Group differences with BD and HCs approached significance (*p* values ≤ 0.062).

### Potential covariates

Key variables for which unplanned group differences emerged were evaluated as potential covariates for primary statistical models on an ROI-by-ROI basis. Per Table 1, there were factor effects for BD, AUD, and/or the BD × AUD interaction (*p* values < 0.10) that warranted the evaluation of age, manic (YMRS) and depressive (MADRS) symptoms, antipsychotic medication use, past-month drinking, and anxiety disorder(s) for effects on primary seed-based connectivity outcomes. Specific covariates that had significant effects on ROI outcomes, including age (right IFG), past-month drinking (right dAI), past 3-month anxiety disorders (right caudate), manic symptoms, and antipsychotic medication use (left caudate), were retained for their respective statistical models.

### Cue-modulated functional connectivity

**Fig. 1:**
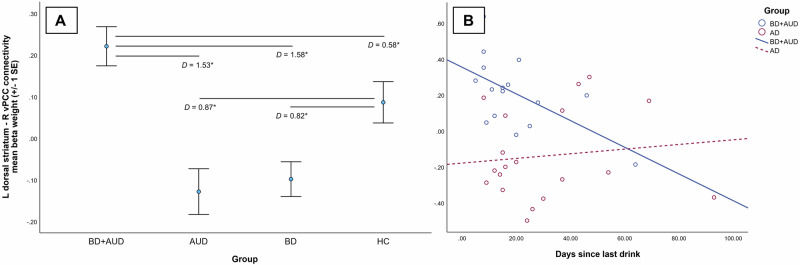
Dorsal striatal-PCC coupling in BD + AUD and associations with duration of abstinence. **A** Data are mean beta weight values by group (±1 standard error bars) with effect sizes in Cohen’s *D*. **B** Data are bivariate correlations with linear regression lines fitted by group. BD + AUD, *R*^2^ = 0.34; AUD, *R*^2^ = 0.01; BD, *R*^2^ = 0.03; HC, *R*^2^ = 0.03. vPCC ventral posterior cingulate córtex, L left, R right. *p ≤ 0.05.

**Fig. 2:**
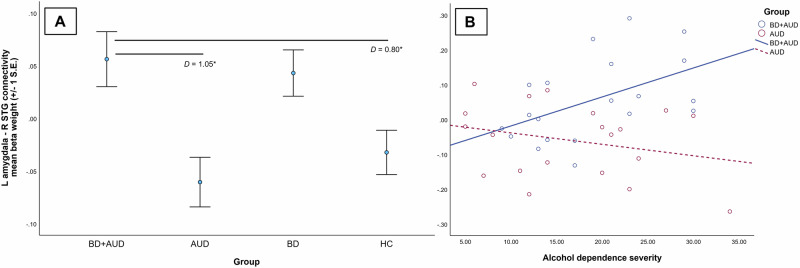
Amygdala-STG coupling in BD + AUD and associations with alcohol dependence severity. **A** Data are mean beta weight values by group (±1 standard error bars) with effect sizes in Cohen’s *D*. **B** Data are bivariate correlations with linear regression lines fitted by group. BD + AUD, *R*^2^ = 0.25; AUD, *R*^2^ = 0.07. STG superior temporal gyrus, L left, R right; alcohol dependence severity per ADS scores. *p* ≤ 0.05.

**Fig. 3:**
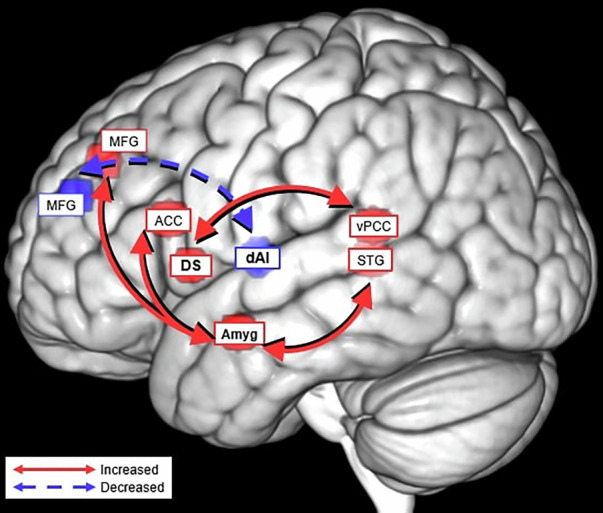
Aberrant cue-modulated functional connectivity in BD + AUD. Task-based functional connectivity in BD+AUD versus HC participants. Seeds (ROIs) in bold: dorsal striatum (DS [left]; caudate body), dorsal anterior insula (dAI [right]), and amygdala (amyg [left]). Targets: MFG middle frontal gyrus, ACC anterior cingulate cortex, vPCC ventral posterior cingulate cortex, STG superior temporal gyrus.

Seed-based gPPI analyses revealed significant factor effects on cue-modulated functional connectivity for ROIs including the left dorsal striatum (BD × AUD interaction, *p*-FDR = 0.045), the left amygdala (BD main effect, *p*-FDR ≤ 0.040), and the right dAI (AUD main effect, *p*-FDR = 0.001). Specifically, we found individuals with BD + AUD to exhibit left dorsal striatal ↔ right ventral PCC hyperconnectivity during cue processing relative to healthy control participants (*p* = 0.051) and individuals with AUD or BD alone (*p* values < 0.001), who in turn exhibited hypoconnectivity between these regions relative to both BD + AUD and HC participants (*p* values ≤ 0.007) (Fig. 1A). Participants with BD + AUD and BD alone both exhibited left amygdala hyperconnectivity with the left ventral ACC and contralateral middle frontal gyrus, superior temporal gyrus (Fig. 2A) and precuneus, relative to AUD alone and healthy control groups (*p* values ≤ 0.048). Finally, participants with BD + AUD and AUD both exhibited right dAI hypoconnectivity with the ipsilateral middle frontal gyrus relative to BD alone and healthy control groups (*p* values ≤ 0.005). For the resulting pattern of cue-modulated functional connectivity in BD + AUD, see Fig. 3. For gPPI betas and group differences, see Table 2.

**Table 2:** Significant regions of interest and gPPI whole-brain cluster beta values by participant groupings.

Region of interest	Factor effect	Cluster (*p*-FDR)	*k*	Center coordinates	Participant group			
					BD + AUD^1^ (*n* = 20)	AUD^2^ (*n* = 20)	BD^3^ (*n* = 22)	HC^4^ (*n* = 25)
Dorsal striatum (L)	BD × AUD interaction	Posterior cingulate cortex	77	4, −38, 16	0.22 (0.21)^2,3^	−0.12 (0.25)^1,4^	−0.10 (0.20)^1,4^	0.09 (0.25)^2,3^
Dorsal anterior insula (R)	AUD main effect	Middle frontal gyrus	227	28, 44, 22	−0.09 (0.30)^3,4^	−0.15 (0.20)^3,4^	0.18 (0.26)^1,2^	0.13 (0.16)^1,2^
Amygdala (L)	BD main effect	Anterior cingulate cortex	65	−04, 24, 18	0.06 (0.08)^2,4^	−0.07 (0.10)^1,3^	0.08 (0.11)^2,4^	−0.02 (0.10)^1,3^
		Middle frontal gyrus	98	38, 50, 34	0.07 (0.09)^2,4^	−0.07 (0.07)^1,3^	−0.03 (0.11)^2,4^	−0.03 (0.11)^1,3^
		Superior temporal gyrus	69	56, −30, 4	0.06 (0.12)^2,4^	−0.06 (0.11)^1,3^	0.04 (0.10)^2,4^	−0.03 (0.11)^1,3^
		Precuneus	53	14, −74, 38	0.60 (0.13)^2,4^	−0.02 (0.08)^1^	0.04 (0.14)^4^	−0.11 (0.14)^1,3^

Superscripts denote groups and group differences (*p* < 0.05).

*L* left, *R* right, *k* number of voxels.

### Behavioral correlates

Potential associations between cue-modulated functional connectivity data and behavioral correlates of interest, including clinical characteristics of AUD (e.g., age of AUD onset, alcohol craving, etc.), were primarily examined across and within AUD groups due to restricted range within BD and HC groups. Of the analyses performed, significant brain-behavior associations were exclusively found in BD + AUD participants, whereby greater left dorsal striatal ↔ right ventral PCC functional connectivity was associated with a fewer number of days since last drink (*r* = 0.59, *p* = 0.017; Fig. 1B), and greater left amygdala ↔ right superior temporal gyrus functional connectivity was associated with both greater alcohol dependence severity (*r* = 0.50, *p* = 0.026; Fig. 2B) and an earlier age of onset for AUD (*r* = −0.53, *p* = 0.020). See Tables S2–S4 for correlation matrices.

## Discussion

We found a unique pattern of aberrant cortico-striato-limbic functional connectivity to characterize alcohol-cue processing in BD + AUD. Relative to our respective comparison-control groups (i.e., BD, AUD, and HC participants), individuals with BD + AUD exhibited contralateral cue-modulated hyperconnectivity between the dorsal striatum (i.e., caudate body) and ventral PCC, and between the amygdala and superior temporal gyrus, relative to AUD and HC participants, which were in turn associated with clinical features of AUD (e.g., an earlier age of AUD onset, greater alcohol dependence severity, and a shorter duration of sustained abstinence, respectively) in BD + AUD but not AUD. Additionally, BD + AUD individuals exhibited cue-modulated hypoconnectivity between the right dAI and the ipsilateral middle frontal gyrus, suggesting salience network dysfunction. These novel findings, conceptualized to represent aspects of abnormal reward-stimulus learning, disinhibition, and relapse vulnerability/protective processes in BD + AUD, could serve as high-yield targets for treatment innovation.

We previously found individuals with BD + AUD, relative to the same comparison-control groups, to uniquely exhibit cue-elicited hypoactivations within the dorsal striatum and a whole-brain cluster spanning portions of the right dAI and adjacent IFG, which, in turn, were associated with higher levels of craving and impulsivity-related features exclusively in BD + AUD [27]. Here, we found individuals with BD + AUD to exhibit cue-modulated hyperconnectivity between the left dorsal striatum and right PCC relative to AUD and BD groups, who both exhibited hypoconnectivity between these regions relative to HC participants. Previous studies have also found PCC hypoconnectivity in response to cues in AUD, potentially reflecting PCC-generated electrophysiological effects triggered by craving and cue processing interactions [66–68] and during resting-state in BD [69], which, given that many BD participants had not drank alcohol in years, may have been approximated (via task disengagement) in the present study. Meta-analytic cognitive modeling and structural neuroimaging findings have shown that the caudate is strongly connected to action and perception brain networks involving the PCC [70], which itself is involved in internally directed attention (e.g., introspective/interoceptive processes) and is integral to adapting and strategizing [68] motivated behavior. In light of our correlational findings, dorsal striatal ↔ ventral PCC hyperconnectivity in BD + AUD may represent a distinct state-like mechanism during initial periods of sustained abstinence, indicating a heightened degree of self-referencing (e.g., default-mode network activation) and action planning (e.g., the pursuit of alcohol) in the presence of cues [69–73] thereby increasing relapse vulnerability [67, 74, 75]. Alternatively, dorsal striatal ↔ ventral PCC hyperconnectivity in BD + AUD could also be adaptive, reflecting heightened degrees of motivation and cognitive effort to maintain abstinence. Hypoconnectivity between these regions in AUD alone may also represent a state-like mechanism, but the lack of correlational findings with behavioral data limits interpretability. Regardless of directionality, pharmacological interventions that target dopaminergic neurotransmission (e.g., D2/3 [ant]agonists; [76]) with the capacity to systemically modulate dorsal striatal circuitry, as has been demonstrated in healthy samples [77], may be particularly beneficial for patients in the early stages of recovery from AUD.

We otherwise found AUD and BD to exert independent effects on cue-modulated functional connectivity. Participants with BD (i.e., BD + AUD and BD groups) exhibited cue-modulated amygdala hyperconnectivity with principal nodes of the salience network (ACC, middle frontal gyrus [i.e., frontal pole]) and the superior temporal gyrus. Spatial processing and stimulus discrimination, in this case between alcohol versus non-alcohol beverage images, both involve the superior temporal gyrus ─ positively-valenced modulation by the amygdala may enhance and/or accelerate alcohol-cue conditioning in individuals with BD [35, 78, 79]. Supporting this notion, we found greater amygdala ↔ superior temporal gyrus functional connectivity to be associated with greater alcohol dependence severity and an earlier age of onset for AUD, exclusively among individuals with BD + AUD. Separately, we found participants with AUD (i.e., BD + AUD and AUD groups) to exhibit hypoconnectivity between the right dAI and middle frontal gyrus (i.e., anterior PFC), a circuit disruption that has elsewhere been associated with emotion dysregulation in BD and behavioral inflexibility (i.e., impulsive/compulsive alcohol use) in AUD [25, 80, 81]. Taken together in BD + AUD, hyperactive cortico-amygdala circuitry, corresponding with heightened emotional reactivity (and impulsivity) [82], may influence associative learning and incentive salience mechanisms and/or broadly undermine motivated behavior by disrupting the salience network’s capacity to facilitate the dynamic neural communication (i.e., “toggling” between other brain networks) required for adaptive reward decision-making. Impaired top-down anterior prefrontal modulation of the dAI may result in amplified interoceptive awareness that exacerbates craving-related motivational states and contributes to cue-elicited disinhibition in BD + AUD. In translation, leveraging neuromodulation techniques like repetitive transcranial magnetic stimulation (rTMS) that can effectively normalize the functioning of the salience network (e.g., by targeting the amygdala indirectly via the ventrolateral prefrontal cortex; [83]) and the default-mode network (e.g., by targeting the PCC; [84]), in reference to protocols that are successfully being developed for other severe dual diagnosis populations [85], would represent a novel path towards advanced treatment for BD + AUD.

Whether the aberrant reward-related functional connectivity we identified in BD + AUD plays a causal role and/or is a consequence of disorder onset (e.g., resulting from alcohol-induced neurotoxicity, neurosensitization from recurrent mood episodes, prolonged psychotropic medication use, etc.) remains to be determined. Familial/high-risk neuroimaging studies to date examining reward circuitry functioning among non-symptomatic children and adolescents of parents with BD, in an effort to identify vulnerability biomarkers, have found that these predisposed youth exhibit abnormal (para)limbic and cortico-striatal reward-modulated functional connectivity, suggesting they encode anticipated rewards with greater incentive salience (e.g., ↑ ventral striatum ↔ insula) that is capable of triggering poorly-inhibited affective and motivational states (e.g., ↓ pregenual ACC ↔ ventrolateral prefrontal cortex) consistent with emotion dysregulation and mood lability characteristic of emerging BD [86, 87]. Similar familial/high-risk studies of youth predisposed to AUD suggest that ventral striatal hyperconnectivity with cognitive control regions (e.g., IFG) may reflect a pronounced functional imbalance favoring the reward network during adolescence, creating an ultra-vulnerable period for alcohol misuse and the development of alcohol-related problems. In parallel, and in light of the present study findings, emerging evidence suggests that deficient inhibitory control and anomalous reward functioning likely exist as both contributing risk factors and consequences of BD + AUD [88–90].

The present study had many strengths, including the study design, well-characterized clinical samples, and statistical rigor, but was not without limitations. Recruitment and retention of individuals with BD + AUD for research is notoriously difficult [4]. Our sample sizes were relatively small, which likely limited our statistical power and resulted in some undetected true effects. Yet with well-characterized samples, the significant group effects we did identify may have more conceptual and clinical utility. Regardless, larger-scale replication studies are warranted. Despite rigorous recruitment efforts to attain strictly matched samples, some unanticipated group differences emerged that could have influenced findings; for instance, anxiety disorders were less prevalent among AUD participants (versus BD + AUD and BD participants) than anticipated. Functional connectivity, by definition, is correlational and not a measure of the direct influence that one brain region exerts over another, thereby limiting the specificity of inferences made from these data [91, 92]. Given that our prior cue reactivity (i.e., functional activation) findings, obtained from this same sample [27], directly informed the ROIs we investigated, it is possible that selection bias improved our ability to detect significant effects. Finally, causal inferences are limited given the cross-sectional study design. Future comprehensive longitudinal research is needed to establish causal mechanisms and reliable biomarkers for BD + AUD.

In conclusion, we found BD + AUD to be distinguished by a unique pattern of aberrant cue-modulated cortico-striato-limbic functional connectivity that was further contextualized by distinct brain-behavior relationships involving candidate vulnerability and maintenance mechanisms. Establishing causal effects (i.e., effective connectivity) between the regional and circuit-level disruptions we identified would represent a natural extension of these findings and help prioritize neural targets for testing promising investigative treatments (e.g., rTMS) for BD + AUD.

## Supplementary information

Supplementary Materials

## Data Availability

Please contact the corresponding author, JJP, for data available upon request.
