# Supplementary material for: Aberrant cortico-striato-limbic functional connectivity to alcohol cues in co-occurring bipolar disorder and alcohol use disorder
Source: Neuropsychopharmacology. 2026 May 2;51(8):1378–86. doi: 10.1038/s41386-026-02418-x (PMC13291348; doi:10.1038/s41386-026-02418-x)
Supplement: Supplementary file 1 — Supplementary Materials [file 41386_2026_2418_MOESM1_ESM.docx]

**Supplemental Materials**

*Denoising.* fMRI data were denoised using a standard denoising pipeline including the regression of potential confounding effects characterized by white matter timeseries and their first order derivatives (96 CompCor noise components), CSF timeseries (48 CompCor noise components), motion parameters and their first order derivatives (12 factors), outlier scans (below 160 factors), session and task effects and their first order derivatives (10 factors) and linear trends (2 factors), followed by bandpass frequency filtering of the BOLD timeseries above 0.008 Hz (Nieto-Castanon, 2020). CompCor (Hallquist et al., 2023) noise components within white matter and CSF were estimated by computing the average BOLD signal as well as the largest principal components orthogonal to the BOLD average, motion parameters, and outlier scans within each subject’s eroded segmentation masks. From the number of noise terms included in this denoising strategy, the effective degrees of freedom of the BOLD signal after denoising were estimated to range from 0 to 154.4 (average 144.1) across all subjects. Quality control procedures resulted in a total of 22 participants being removed from the overall sample (BD+AUD, *n* = 6; AUD, *n* = 6; BD, *n* = 8; HC, *n* = 2), 17 of which were identified as outliers using our optimal selected threshold of ≥ 5% for the number of invalid scans.

*Quality Control Pipeline.* Quality control procedures for structural and functional brain data were implemented following best practice recommendations by the CONN toolbox development team (Morfini et al., 2023). Participant-level QC procedures included visual inspection of raw-level data and of representative images after each preprocessing step, as well as the computation of automated descriptive QC measures including but not limited to average framewise displacement (FD), mean global signal change (MeanGSchange), and prevalence of outlier scans. Dataset-level QC procedures included the evaluation of inter-subject variability in the distributions of edge connectivity in a 1,000-node graph (FC distribution displays), and the estimation of residual associations across participants between functional connectivity strength and potential noise indicators such as participant’s head motion and prevalence of outlier scans (QC-FC analyses).QC indices included but were not limited to MeanGSchange and Mean Motion, representing GSC or FD values limited only to valid (non-outlier) scans, along with the number of invalid (outlier) scans/proportion of valid scans (PVS), and quantification of spatial normalization performance, providing several indicators of the overall quality and amount of valid data within the run for each subject. Lastly, the QC measure QC-FC% (percent match in QC-FC correlations) characterizing properties of the entire dataset, were evaluated to ensure preprocessing, denoising, and outlier identification steps resulted in satisfactory data quality. To obtain the recommended 95% QC-FC% match level for key QC indices (e.g., mean motion, invalid scans/PVS, and meanGSchange; Morfini et al., 2023) we iteratively re-evaluated QC-FC correlations, excluding one additional participant at a time, to identify the minimal number of excluded participants to achieve an optimal balance between data quality and data retention.

Our QC procedures resulted in a total of 22 participants being removed from the overall sample (BD+AUD, *n* = 6; AUD, *n* = 6; BD, *n* = 8; HC, *n* = 2). Seventeen of these participants were identified as outliers using our (optimal) selected threshold of ≥ 5% for number of invalid scans (BD+AUD, *n* = 3; AUD, *n* = 5; BD, *n* = 7; HC, *n* = 2) and the remaining 5 were excluded for structural or functional normalization performance (BD+AUD, *n* = 3; AUD, *n* = 1; BD, *n* =1). Omitting these cases yielded QC-FC% match levels that approached if not exceeded the desired 95% threshold, e.g., for: number of invalid scans/PVS = 95.3% (before denoising = 82.3%), mean motion = 94.7% (before denoising = 68.5%; max motion = 96.1%), meanGSchange = 97.2% (before denoising = 91.5%), and the performance of spatial normalization (functional/structural) ≥ 98.2% (before denoising ≤ 85.1%).

**References**

Hallquist, M. N., Hwang, K., & Luna, B. (2013): The nuisance of nuisance regression: spectral misspecification in a common approach to resting-state fMRI preprocessing reintroduces noise and obscures functional connectivity. *Neuroimage* 82: 208-225.

Nieto-Castanon, A. (2022): Brain-wide connectome inferences using functional connectivity MultiVariate Pattern Analyses (fc-MVPA). *PLoS computational biology* 18(11): 1-28.

Morfini, F., Whitfield-Gabrieli, S., & Nieto-Castañón, A. (2023): Functional connectivity MRI quality control procedures in CONN. *Frontiers in Neuroscience* 17: 1-22.

**Figure S1.** Alcohol cue-reactivity paradigm: Representative pictures and time course

| 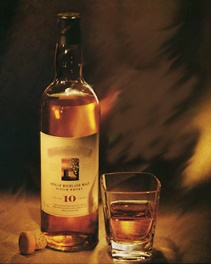 | 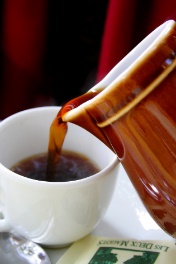 | 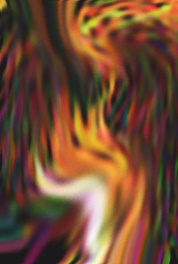 | 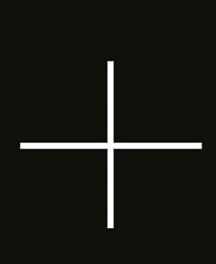 | 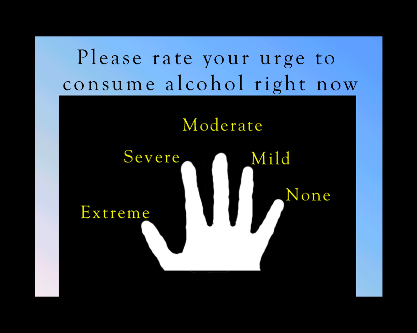 |
| --- | --- | --- | --- | --- |
| **Alcohol (A)** | **Beverage (B)** | **Blur (C)** | **Fixation (D)** | **Rating** |

| B | C | A |  | C | B |  | A | C | B | A |  | A | C | B |  | B |  | C | A |  | B | A | C |
| --- | --- | --- | --- | --- | --- | --- | --- | --- | --- | --- | --- | --- | --- | --- | --- | --- | --- | --- | --- | --- | --- | --- | --- |
|  |  |  | D |  |  | D |  |  |  |  | D |  |  |  | D |  | D |  |  | D |  |  |  |
|  | | | | | | | | | | | | | | | | | | | | | | | |

Total duration of 12 minutes including six 120s epochs consisting of three 24s blocks of an image type (one block each of alcohol, non-alcohol beverage, blur, and fixation) presented in pseudorandom order. Each category block consists of 5 pictures (4.8s/picture) followed by an “urge to drink alcohol” handpad rating (6s).

**Table S1.** Descriptive statistics and group comparison results for behavioral correlates examined

|  | **BD+AUD**^1^  (*n* = 22) | **AUD**^2^  (*n* = 20) | **BD**^3^  (*n* = 23) | **HC**^4^  (*n* = 25) | ***F*(*p*)** | | |
| --- | --- | --- | --- | --- | --- | --- | --- |
|  |  |  |  |  | **AUD** | **BD** | **BD x AUD** |
| BIS-11 | 75.70(14.06)^2-4^ | 61.10(12.80)^1,4^ | 67.35(16.30)^1,4^ | 48.12(6.85)^1-3^ | 15.05(<.001) | 37.83(<.001) | 0.71(0.402) |
| Attentional^*^ | 19.75(4.12)^2-4^ | 14.90(4.22)^1,3,4^ | 17.70(5.51)^1^ | 10.46(2.06)^1-3^ | 13.26(<.001) | 45.92(<.001) | 1.79(0.184) |
| Motor^*^ | 26.30(5.48)^2,4^ | 22.35(4.30)^1,4^ | 24.00(5.81)^4^ | 18.04(2.53)^1-3^ | 10.60(0.002) | 23.84(<.001) | 0.98(0.326) |
| Non-planning | 29.65(6.26)^2-4^ | 23.85(6.12)^1,4^ | 25.65(6.73)^1,4^ | 18.64(3.23)^1-3^ | 14.36(<.001) | 27.79(<.001) | 0.25(0.619) |
| Days Since Last  Drink^*^ | 19.50(15.66) | 30.79(22.50)^4^ | 27.39(22.86)^4^ | 14.67(6.26)^2,3^ | 0.97(0.329) | 0.03(0.865) | 8.23(0.005) |
| MAXdrinks | 22.87(10.04)^3,4^ | 25.50(9.52)^3,4^ | 8.11(5.09)^1,2^ | 8.80(4.07)^1,2^ | 92.25(<.001) | 1.03(0.314) | 0.35(0.554) |
| ADS | 19.00(6.94)^3,4^ | 16.70(8.58)^3,4^ | 2.09(2.15) | 0.72(1.34) | 201.39(<.001) | 2.51(0.117) | 0.16(0.689) |
| OCDS | 9.70(7.13)^2-4^ | 4.70(3.70)^1,3,4^ | 0.74(1.14)^1,2^ | 0.96(0.84)^1,2^ | 58.07(<.001) | 8.22(0.005) | 9.81(0.002) |
| Compulsive | 5.55(4.10)^2-4^ | 2.50(2.74)^1,3,4^ | 0.61(0.94)^1,2^ | 0.88(0.78)^1,2^ | 39.73(<.001) | 7.12(0.009) | 10.18(0.002) |
| Obsessive^*^ | 4.15(3.30)^2-4^ | 2.20(1.44)^1,3,4^ | 0.00 (0.00)^1,2^ | 0.00(0.00)^1,2^ | 68.69(<.001) | 6.45(0.013) | 6.48(0.013) |

Data are mean(S.D.) and *F*-value(*p-*value), respectively. Superscripts^1-4^ assigned to descriptive statistics denote significant group differences (uncorrected *p* < 0.05). AUD = alcohol use disorder; BD = bipolar disorder; BIS-11= Barratt Impulsiveness Scale; Days Since Last Drink = number of days since last alcohol drink before MRI; MAXdrinks = lifetime maximum number of alcohol drinks consumed within a 24-hour period; OCDS = Obsessive-Compulsive Drinking Scale. %HDD-30 = percent of past 30 days defined as heavy drinking days (> 4 drinks/day for men, > 3 drinks/day for women)

*Extreme outliers for days since last drink were removed from the BD (*n* = 2) and HC (*n* = 3) groups. Without extreme outlier removal, the BD x AUD interaction was non-significant (*F* = 1.63, *p* = 0.206) and the group mean(SD) were: BD = 317.20(1009.84) and HC = 32.92(54.27). Extreme outliers were also removed for OCDS Obsessive, BIS-11 Attentional, and BIS-11 Motor scores. Without extreme outlier removal, there was minimal change in OCDS Obsessive (BD, *n* = 2 [0.13(0.46)]; HC, *n* = 2 [0.08(0.28)]), BIS-11 Attentional (HC, *n* = 1 [10.80(2.65)]), and BIS-11 Motor (HC, *n* = 2 [18.68(3.28)]) scores and the significance of factor effects and group differences were consistent.

**Table S2.** Correlations between key variables within the BD+AUD group

|  | **1** | **2** | **3** | **4** | **5** | **6** | **7** | **8** | **9** | **10** | **11** | **12** | **13** | **14** | **15** | **16** |
| --- | --- | --- | --- | --- | --- | --- | --- | --- | --- | --- | --- | --- | --- | --- | --- | --- |
| 1. Caudate ↔ PCC | - |  |  |  |  |  |  |  |  |  |  |  |  |  |  |  |
| 2. dAI ↔ MFG | .19 (.443) | - |  |  |  |  |  |  |  |  |  |  |  |  |  |  |
| 3. Amyg ↔ STG | -.54 (.015)* | -.04 (.886) | - |  |  |  |  |  |  |  |  |  |  |  |  |  |
| 4. Amyg ↔ MFG | -.53 (.015)* | -.01 (.978) | .44 (.051) | - |  |  |  |  |  |  |  |  |  |  |  |  |
| 5. Amyg ↔ ACC | -.12 (.629) | .00 (.997) | .21 (.375) | .10 (.662) | - |  |  |  |  |  |  |  |  |  |  |  |
| 6. Amyg ↔ PreC | -.61 (.004)* | -.13 (.601) | .56 (.010)* | .48 (.033)* | -.11 (.656) | - |  |  |  |  |  |  |  |  |  |  |
| 7. OCDS Total | -.03 (.894) | -.14 (.573) | .01 (.955) | .14 (.560) | .03 (.914) | .15 (.525) | - |  |  |  |  |  |  |  |  |  |
| 8. OCDS Obsessive | -.13 (.581) | -.16 (.516) | -.22 (.364) | .25 (.294) | .00 (.989) | .19 (.423) | .96 (<.001)* | - |  |  |  |  |  |  |  |  |
| 9. OCDS Compulsive | .05 (.833) | -.12 (.644) | .13 (.590) | .04 (.857) | .05 (.842) | .11 (.644) | .97 (<.001)* | .86 (<.001)* | - |  |  |  |  |  |  |  |
| 10. ADS | -.44 (.050)* | -.10 (.700) | -.08 (.737) | .32 (.172) | .01 (.969) | .21 (.375) | .07 (.776) | .17 (.473) | -.02 (.938) | - |  |  |  |  |  |  |
| 11. MAXdrinks | -.33 (.175) | .37 (.148) | .50 (.026)* | .02 (.931) | -.27 (.271) | .02 (.949) | -.15 (.542) | -.01 (.961) | -.25 (.297) | .72 (<.001)* | - |  |  |  |  |  |
| 12. DSLD | -.59 (.017)* | -.16 (.563) | .40 (.088) | .15 (.571) | -.15 (.592) | .28 (.289) | -.54 (.030)* | -.42 (.107) | -.61 (.013)* | .39 (.144) | .30 (.255) | - |  |  |  |  |
| 13. BIS-11 Total | -.05 (.826) | -.20 (.434) | .08 (.724) | .12 (.604) | .11 (.659) | -.16 (.489) | .15 (.530) | .23 (.331) | .08 (.753) | .53 (.017)* | .39 (.097) | .20 (.457) | - |  |  |  |
| 14. BIS-11 Attentional | -.13 (.593) | -.13 (.604) | -.01 (.968) | .10 (.676) | .04 (.853) | .00 (.994) | .28 (.228) | .33 (.162) | .23 (.329) | .39 (.092) | .30 (.220) | -.03 (.900) | .88 (<.001)* | - |  |  |
| 15. BIS-11 Motor | .19 (.427) | -.08 (.766) | .01 (.954) | -.11 (.642) | -.04 (.874) | -.38 (.103) | .06 (.818) | .08 (.731) | .03 (.901) | .52 (.020)* | .50 (.031)* | .06 (.834) | .89 (<.001)* | .73 (<.005)* | - |  |
| 16. BIS-11 Non-planning | -.20 (.400) | -.28 (.259) | .18 (.439) | .31 (.185) | .24 (.308) | -.04 (.861) | .10 (.671) | .23 (.330) | -.01 (.972) | .48 (.032)* | .26 (.287) | .40 (.122) | .89 (<.001)* | .69 (<.001)* | .64 (.003)* | - |

PCC = posterior cingulate cortex; dAI = dorsal anterior insula; Amyg = amygdala; STG = superior temporal gyrus; MFG = middle frontal gyrus; ACC = anterior cingulate cortex; PreC = precuneus; OCDS = Obsessive-Compulsive Drinking Scale (with obsessive and compulsive subscales); ADS = alcohol dependence severity; MAXdrinks = Maximum number of alcohol drinks consumed on a drinking day; DSLD = Number of days since last alcohol drink consumed; BIS-11 = Barratt Impulsiveness Scale (with Attentional, Motor, and Non-planning subscales) **p* ≤ 0.05

**Table S3.** Correlations between key variables within the AUD group

|  | **1** | **2** | **3** | **4** | **5** | **6** | **7** | **8** | **9** | **10** | **11** | **12** | **13** | **14** | **15** | **16** |
| --- | --- | --- | --- | --- | --- | --- | --- | --- | --- | --- | --- | --- | --- | --- | --- | --- |
| 1. Caudate ↔ PCC | - |  |  |  |  |  |  |  |  |  |  |  |  |  |  |  |
| 2. dAI ↔ MFG | -.33 (.169) | - |  |  |  |  |  |  |  |  |  |  |  |  |  |  |
| 3. Amyg ↔ STG | -.05 (.849) | .07 (.766) | - |  |  |  |  |  |  |  |  |  |  |  |  |  |
| 4. Amyg ↔ MFG | -.11 (.656) | -.06 (.807) | -.05 (.828) | - |  |  |  |  |  |  |  |  |  |  |  |  |
| 5. Amyg ↔ ACC | -.07 (.764) | .33 (.171) | .15 (.534) | -.17 (.482) | - |  |  |  |  |  |  |  |  |  |  |  |
| 6. Amyg ↔ PreC | .19 (.415) | .08 (.755) | -.06 (.805) | -.28 (.231) | .38 (.097) | - |  |  |  |  |  |  |  |  |  |  |
| 7. OCDS Total | -.16 (.504) | .09 (.722) | -.01 (.974) | -.35 (.131) | .23 (.321) | .08 (.751) | - |  |  |  |  |  |  |  |  |  |
| 8. OCDS Obsessive | .06 (.788) | .08 (.756) | -.22 (.364) | -.25 (.290) | -.01 (.971) | -.13 (.575) | .78 (<.001)* | - |  |  |  |  |  |  |  |  |
| 9. OCDS Compulsive | -.25 (.292) | .08 (.751) | .10 (.669) | -.34 (.141) | .32 (.169) | .17 (.469) | .94 (<.001)* | .52 (.019)* | - |  |  |  |  |  |  |  |
| 10. ADS | -.48 (.034)* | .13 (.599) | -.27 (.255) | -.05 (.829) | .19 (.415) | -.06 (.804) | .18 (.437) | .25 (.282) | .12 (.625) | - |  |  |  |  |  |  |
| 11. MAXdrinks | -.04 (.886) | .10 (.680) | -.16 (.527) | .29 (.231) | .05 (.829) | -.32 (.175) | .20 (.421) | .51 (.027) | .00 (.997) | .45 (.056) | - |  |  |  |  |  |
| 12. DSLD | .12 (.631) | -.33 (.167) | .11 (.647) | -.27 (.260) | -.09 (.727) | -.06 (.816) | -.49 (.033)* | -.41 (.081) | -.45 (.056) | -.14 (.572) | -.36 (.142) | - |  |  |  |  |
| 13. BIS-11 Total | -.10 (.671) | -.09 (.704) | -.48 (.033)* | -.17 (.478) | -.09 (.697) | -.45 (.046)* | .45 (.045)* | .49 (.030)* | .36 (.121) | .10 (.665) | .13 (.596) | .07 (.769) | - |  |  |  |
| 14. BIS-11 Attentional | .04 (.876) | -.13 (.598) | -.45 (.046)* | -.34 (.143) | -.26 (.261) | -.40 (.079) | .41 (.070) | .46 (.044)* | .32 (.171) | -.13 (.592) | .04 (.885) | .12 (.619) | .90 (<.001)* | - |  |  |
| 15. BIS-11 Motor | -.33 (.163) | .02 (.931) | .39 (.088) | .16 (.505) | -.15 (.519) | -.48 (.034)* | .47 (.038)* | .49 (.028)* | .37 (.105) | .33 (.155) | .42 (.074) | -.23 (.346) | .80 (<.001)* | .61 (.005)* | - |  |
| 16. BIS-11 Non-planning | -.01 (.968) | -.12 (.634) | -.42 (.068) | -.23 (.330) | .10 (.690) | -.34 (.149) | .34 (.145) | .36 (.123) | .27 (.254) | .07 (.762) | -.05 (.840) | .22 (.366) | .91 (<.001)* | .76 (<.001)* | .56 (.011)* | - |

PCC = posterior cingulate cortex; dAI = dorsal anterior insula; Amyg = amygdala; STG = superior temporal gyrus; MFG = middle frontal gyrus; ACC = anterior cingulate cortex; PreC = precuneus; OCDS = Obsessive-Compulsive Drinking Scale (with obsessive and compulsive subscales); ADS = alcohol dependence severity; MAXdrinks = Maximum number of alcohol drinks consumed on a drinking day; DSLD = Number of days since last alcohol drink consumed; BIS-11 = Barratt Impulsiveness Scale (with Attentional, Motor, and Non-planning subscales) **p* ≤ 0.05

**Table S4.** Correlations between key variables within the combined AUD groups

|  | **1** | **2** | **3** | **4** | **5** | **6** | **7** | **8** | **9** | **10** | **11** | **12** | **13** | **14** | **15** | **16** |
| --- | --- | --- | --- | --- | --- | --- | --- | --- | --- | --- | --- | --- | --- | --- | --- | --- |
| 1. Caudate ↔ PCC | - |  |  |  |  |  |  |  |  |  |  |  |  |  |  |  |
| 2. dAI ↔ MFG | -.11 (.520) | - |  |  |  |  |  |  |  |  |  |  |  |  |  |  |
| 3. Amyg ↔ STG | .10 (.549) | .08 (.638) | - |  |  |  |  |  |  |  |  |  |  |  |  |  |
| 4. Amyg ↔ MFG | .22 (.177) | .07 (.704) | .48 (.002)* | - |  |  |  |  |  |  |  |  |  |  |  |  |
| 5. Amyg ↔ ACC | .31 (.049)* | .19 (.255) | .41 (.009)* | -.39 (.013)* | - |  |  |  |  |  |  |  |  |  |  |  |
| 6. Amyg ↔ PreC | .03 (.842) | .00 (.997) | .45 (.004)* | .41 (.008)* | .29 (.070) | - |  |  |  |  |  |  |  |  |  |  |
| 7. OCDS Total | .20 (.214) | -.02 (.906) | .20 (.215) | .28 (.082) | .32 (.045)* | .26 (.102) | - |  |  |  |  |  |  |  |  |  |
| 8. OCDS Obsessive | .18 (.259) | -.05 (.779) | .20 (.220) | .33 (.037)* | .22 (.174) | .24 (.139) | .93 (<.001)* | - |  |  |  |  |  |  |  |  |
| 9. OCDS Compulsive | .20 (.224) | .00 (.994) | .19 (.253) | .22 (.178) | .36 (.021)* | .26 (.108) | .97 (<.001)* | .80 (<.001)* | - |  |  |  |  |  |  |  |
| 10. ADS | -.27 (.094) | .02 (.894) | .15 (.352) | .20 (.211) | .18 (.256) | .14 (.403) | .16 (.339) | .22 (.178) | .10 (.548) | - |  |  |  |  |  |  |
| 11. MAXdrinks | -.22 (.184) | .24 (.168) | .08 (.646) | .01 (.963) | -.16 (.352) | -.15 (.376) | -.09 (.536) | .07 (.688) | -.19 (.244) | .53 (<.001)* | - |  |  |  |  |  |
| 12. DSLD | -.26 (.140) | -.25 (.150) | -.03 (.859) | -.23 (.191) | -.24 (.164) | -.06 (.731) | -.52 (.001)* | -.43 (.010)* | -.55 (<.001)* | -.01 (.959) | -.08 (.657) | - |  |  |  |  |
| 13. BIS-11 Total | .25 (.124) | -.08 (.639) | .10 (.538) | .33 (.038)* | .29 (.066) | -.04 (.825) | .39 (.013)* | .41 (.009)* | .34 (.030)* | .33 (.037)* | .17 (.319) | -.02 (.892) | - |  |  |  |
| 14. BIS-11 Attentional | .29 (.069) | -.05 (.770) | .08 (.640) | .29 (.073) | .22 (.169) | .07 (.677) | .46 (.003)* | .46 (.003)* | .41 (.008)* | .16 (.322) | .07 (.666) | -.08 (.639) | .91 (<.001)* | - |  |  |
| 15. BIS-11 Motor | .19 (.230) | .01 (.977) | .06 (.734) | .24 (.129) | .16 (.322) | -.21 (.194) | .30 (.059)* | .29 (.065) | .28 (.080) | .44 (.005)* | .37 (.024)* | -.18 (.293) | .87 (<.001)* | .72 (<.001)* | - |  |
| 16. BIS-11 Non-planning | .20 (.221) | -.14 (.339) | .13 (.431) | .35 (.029)* | .37 (.017)* | .03 (.836) | .32 (.044)* | .37 (.019)* | .26 (.107) | .29 (.067) | .04 (.824) | .14 (.417) | .92 (<.001)* | .78 (<.001)* | .66 (<.001)* | - |

PCC = posterior cingulate cortex; dAI = dorsal anterior insula; Amyg = amygdala; STG = superior temporal gyrus; MFG = middle frontal gyrus; ACC = anterior cingulate cortex; PreC = precuneus; OCDS = Obsessive-Compulsive Drinking Scale (with obsessive and compulsive subscales); ADS = alcohol dependence severity; MAXdrinks = Maximum number of alcohol drinks consumed on a drinking day; DSLD = Number of days since last alcohol drink consumed; BIS-11 = Barratt Impulsiveness Scale (with Attentional, Motor, and Non-planning subscales) **p* ≤ 0.05
